# Renal survival in hereditary urolithiasis: a monocentric cohort study from a Tunisian nephrology department

**DOI:** 10.11604/pamj.2026.53.75.45171

**Published:** 2026-02-10

**Authors:** Asma Bettaieb, Meriam Hajji, Hayet Kaaroud, Fethi Ben Hamida, Rym Goucha, Ridha Mrad, Tahar Gargah, Marouen Chakroun, Kahena Bouzid, Ezzeddine Abderrahim

**Affiliations:** 1 Faculty of Medicine of Tunis, University of Tunis El Manar, Tunis, Tunisia,; 2 Kidney Pathology Laboratory LR00SP01, Charles Nicolle Hospital, Tunis, Tunisia,; 3 Department of Nephrology, Mongi Slim Hospital, Tunis, Tunisia,; 4 Department of Medicine, A-Charles Nicolle Hospital, Tunis, Tunisia,; 5 Department of Genetics, Charles Nicolle Hospital, Tunis, Tunisia,; 6 Department of Pediatrics, Charles Nicolle Hospital, Tunis, Tunisia,; 7 Department of Urology, Charles Nicolle Hospital, Tunis, Tunisia,; 8 Department of Clinical Biochemistry, Charles Nicolle Hospital, Tunis, Tunisia,; 9 Laboratory of Neurophysiology Cellular Physiopathology and Biomolecules Valorisation, University of Tunis El Manar, Tunis, Tunisia

**Keywords:** Hereditary urolithiasis, hyperoxaluria, cystinuria, tubulopathies

## Abstract

**Introduction:**

hereditary urolithiasis (HUL) is rare and often underdiagnosed. The genetic type contributes, with other factors, to the onset of renal failure and its progression to end-stage renal disease (ESRD). The aim of our study was to determine the incidence of progression to ESRD and to identify factors influencing renal survival.

**Methods:**

we conducted an analytical retrospective study in our department of nephrology for 31 years [1990-2021]. Records of patients with HUL who were not in ESRD at diagnosis were retained. We performed a Cox proportional hazards model.

**Results:**

we included 73 patients, of whom 60.3% (n=44) were males. The mean age at the onset of urolithiasis symptomatology was 19.15± 15.57 years. The causes were cystinuria, hereditary tubulopathies and primary hyperoxaluria (PH), in 31, 24 and 18 cases, respectively. The delay in the etiological diagnosis was over 5 years in 41.1% (n=30). After a follow-up of an average period of 16 years, the annual incidence of ESRD was 0.94% and the annual decline in glomerular filtration rate was 3.5 ml/min. The multivariate study retained only PH as an independent factor associated with the occurrence of ESRD with an adjusted hazard ratio of 10.52, 95% CI: 1.664-66.507; p= 0.012. Primary hyperoxaluria had the highest annual incidence of ESRD during follow-up (3.42% vs. 0.46% for tubulopathies). The annual decline in glomerular filtration rate in PH was 5.73 ml/min, vs 2.9 ml/min in cystinuria and 2.67 ml/min in tubulopathies.

**Conclusion:**

primary hyperoxaluria was the main determinant of ESRD, highlighting the need for early etiological and genetic diagnosis, especially in consanguineous populations.

## Introduction

Hereditary urolithiasis (HUL) accounts for only 2% of stones in adults and 10% in children [1]. Moreover, it is most likely that its prevalence is underestimated, particularly in countries characterized by a significant degree of consanguinity, such as Tunisia. There is a significant delay in diagnosing the hereditary nature of HUL in our patients, with an average delay of eight years and a maximum delay of 43 years, to the extent that the key indicators pointing toward a genetic cause were commonly observed [2]. Although several studies suggest that HUL contributes to the onset of renal failure and its progression to end-stage renal disease (ESRD), the mechanisms of this association have not been fully elucidated. Early diagnosis and identification of these factors can avoid or delay such evolution, which increases morbidity and mortality and its socio-economic impact. This study aimed to describe the prognostic features of adult HUL, to determine the incidence of progression of chronic kidney disease (CKD), and to identify factors influencing renal survival.

## Methods

**Study design and setting:** we have conducted an analytical retrospective study in the Internal Medicine A Department of the Charles Nicolle Hospital, which is considered as a leading center in the management of urinary lithiasis in adults in Tunisia, for 31 years [1990-2021]. Records of patients with HUL were retained. The duration of follow-up was determined from the first symptom of urolithiasis and the last consultation before July 1, 2021, or the start date of renal replacement therapy.

**Study population:** the study population consisted of 73 adults hospitalised or seen in consultation and diagnosed with urinary lithiasis of genetic origin. Medical records that were not analyzable due to missing or incomplete data were excluded. Patients were referred to our department for etiological investigation in 41 cases, from paediatric departments to be followed up in adulthood in 23 cases and for chronic renal failure in 9 cases.

**Data collection:** the data were collected retrospectively from patients´ medical records available in the hospital archives. Relevant information was extracted and entered in an anonymized database created in Microsoft Excel for analysis. We specified for the patients: the degree of parental consanguinity, the family history of urinary lithiasis, nephropathy or treatment with extrarenal treatment and death due to chronic renal failure. All patients underwent a biological and radiological investigation, 40 patients had morpho-constitutional stone analysis and 49 patients had crystalluria. The morphological study was carried out by microscopic visualization using a stereomicroscope. Infrared analysis of the stones was performed using a spectrophotometer. The morpho-constitutional classification was based in our study on the classification of Daudon [2]. In the case of primary hyperoxaluria (PH), the genetic study examined only mutations of the AGXT gene for PH type 1 (PH1). The genetic study was performed only in 4 cases for hereditary tubulopathies (HT).

**Definitions:** i) the diagnosis of cystinuria was retained on the morpho-constitutional analysis in the case of the calculi being of Va and/or Vb type or on positive crystalluria; ii) the diagnosis of primary hyperoxaluria (PH) was confirmed by genetic study and/or histopathological features of renal biopsy and morpho-constitutional analysis in the case of the calculi being of Ic type; iii) the diagnosis of hereditary tubulopathies (HT) was based on metabolic assessment. For primary distal tubular acidosis in children, early revelation of the disease in an evocative family context allowed the positive diagnosis. In adults, an etiological investigation was carried out to eliminate the main secondary causes. It included an interrogation and a clinical examination in search of extrarenal signs, a biological assessment (blood count, liver assessment, protein electrophoresis, etc.), a chest X-ray in search of a pleural effusion or parenchymal involvement and an immunological assessment including the determination of antinuclear antibodies and serum complement in all cases. Patients who were in stage 5 of chronic kidney disease (CKD) at the time of diagnosis of HUL, as well as files that could not be used due to lack of data, were excluded. Estimation of eGFR creatinine clearance was made by the MDRD formula for adults and the Schwartz formula for children when height was available [3].

**Statistical analysis:** data were entered and analysed using Excel 365 and SPSS 20.0 software. We calculated the mean annual decline in glomerular filtration rate by relating the difference in the sum of the glomerular filtration rate at the start of follow-up and at the last consultation over the total duration of follow-up in years. Predictors of ESRD were assessed using Cox proportional hazards regression. Variables with p < 0.10 in univariable analysis and those of clinical relevance were entered into the multivariable model. The parameters studied were age, gender, family history of ESRD, delay of diagnosis, recurrence, aetiologies of HUL, occurrence of urinary tract infections or acute kidney injury (AKI), the stage of the CKD at the time of diagnosis, the bilaterality of the calculus, the presence of a coralliform calculus or nephrocalcinosis and the urological treatment. We also calculated ESRD incidence rates by relating the number of patients who started extrarenal purification to the duration of follow-up. The confidence intervals were calculated at 95% by referring to the usual formulas. In all cases, the test was considered significant when the degree of significance p is less than 0.05.

**Ethical considerations:** the study was approved by the local ethics committee of Charles Nicolle Hospital, approval number: FWA 00032748, IORG0011243. Due to the retrospective nature of the study, the requirement for individual informed consent was waived by the committee. All data were anonymized prior to analysis.

## Results

We conducted an analytical retrospective study involving 73 patients to investigate the symptomatology of urolithiasis. We included 73 patients, of whom 60.3% (n=44) were males. Regarding the age-related aspects, the mean age at the onset of urolithiasis symptomatology was 19.15 ± 15.57 years. Additionally, the mean age at which the etiological diagnosis was made was 26.9 ± 17.96 years. The delay in obtaining the etiological diagnosis varied widely, ranging from 0 to 42 years, with a median delay of 3 years. Notably, in 41% (n=30), the diagnosis was made after a delay of more than 5 years. In terms of familial characteristics, we collected information on consanguinity in 64 cases. Among these cases, consanguinity was present in 89% (n=57) of them. Specifically, it was classified as first degree in 65% (n=37) of cases, second degree in 14% (n=8), third degree in 16% (n=9), and distant in 5% (n=3). In 11% cases (n=7), no consanguinity was reported. Sixty-four percent (n=47) reported having an average of 2 affected family members, with the number of affected members ranging from 2 to 5. Out of the total number of families included in the study, 19% (n=14) had at least one family member diagnosed with ESRD. Furthermore, a recurrence of urolithiasis was observed in 64% of cases (n=47) before the initiation of the follow-up period. The aetiologies of HUL were cystinuria, HT and PH in 42% (n=31), 33% (n=24) and 25% (n=18), respectively. Among patients with HT, the underlying causes were distal tubular acidosis in 67% (n=16), hypomagnesemia with familial hypercalciuria in 8% (n=2), hypophosphatemia rickets in 8% (n=2) and idiopathic Toni Debre Fanconi syndrome in 8% (n=2). In addition, 4% (n=1) had hypocalcemia with familial hypercalciuria and another patient had Dent´s disease. At the time of HUL diagnosis, 78% (n=57) of patients had normal renal function and 22% (n=16) had CKD.

Eleven percent (n=8) were at stage 3A, 6% (n=4) at stage 3B and 4% (n=3) at stage 4. The stage of CKD was not specified in 1% (n=1) due to lack of data (height not specified at 8 years of age). The radiological explorations showed that the stone was bilateral in 79% (n=58), coralliform in 15% (n=11) and nephrocalcinosis was noted in 22% (n=16). In the case of PH, a genetic study found the I244T mutation in 12 cases and 33-34 InsC in 2 cases. Mutation in the ATP6V1B1 gene was found in two cases of distal renal tubular acidosis, and a mutation in CLDN16 was found in a case of familial hypomagnesemia with hypercalciuria. Twenty-two percent (n=16) had at least one episode of urinary tract infection during follow-up. Additional infection lithiasis was noted in 5% (n=4) cases. Five percent (n=4) had AKI. The cause was obstructive in one case and post-operative in 3 cases (nephrectomy, appendicular peritonitis and Conn's adenoma). AKI was irreversible in all 4 cases, 2 of which progressed to ESRD. In our study, it was found that 64% (n=47) underwent urological procedures.

These patients had a range of 1 to 8 procedures, with a median of 2 procedures per patient. The different types of urological procedures performed were as follows: extracorporeal lithotripsy (ECL): ECL was performed in 38% of cases (n=28), percutaneous nephrolithotomy (PNL): percutaneous nephrolithotomy was performed in 29% (n=21), endoscopic treatment: endoscopic treatment was performed in 11% of cases (n=8), open surgery: open surgery was performed in 42% (n=31), nephrectomy: nephrectomy, which involves the surgical removal of a kidney, was performed in 5% (n=4). After a median follow-up of 13 years (extremes: 3 months-44 years), 10 patients developed ESRD. The causes were PH in 80% (n=8) and HT in 20 % (n=2): One case was associated with familial hypomagnesemia with hypercalciuria, while the other case was linked to familial hypocalcaemia with hypercalciuria. The age of onset of ESRD ranged from 19 to 45 years with a mean of 31 years. The annual incidence of ESRD was 0.94% for all HUL types [95% confidence interval (CI); 0.36 - 1.52]. The variables that reached significance were history of AKI, nephrectomy, PHO and CKD stages 1 and 2 (Table 1). In the multivariate study, the only independent factor for the occurrence of ESRD was PH, with an adjusted hazard ratio of 10.52, 95% CI 1.664-66.507; p = 0.012 (Table 1). Out of the total number of patients included in the study, data were available to calculate the annual decline in eGFR for 32 patients (44%). It was estimated to be 3.5 ml/min for all HUL types. It was 5.73, 2.9 and 2.67 ml/min for PH, cystinuria and HT, respectively.

**Table 1:** predictors of progression to end-stage renal disease in a monocentric Tunisian cohort of patients with hereditary urolithiasis: results of multivariable Cox proportional hazards regression

			Unajusted HR	P	Adjusted HR	P
Recurrence	Yes		0.799 (0.278-2.295)	0.676	0.431 (0.094-1.974)	0.279
Urinary tract infection	Yes		0.722 (0.178-2.933)	0.648	0.204 (0.021-2.004)	0.173
AKI	Yes		5.682 (1.317-24.507)	0.02	5.511 (0.208-145.720)	0.307
Etiologies	PHO	Yes	6.995 (1.779-27.499)	0.005	10.519 (1.664-66.507)	0.012
Stages of CKD at diagnosis	1+2	Yes	0.223 (0.060-0.831)	0.025	0.457 (0.067-3.112)	0.475
Nephrectomy	Yes		16.193 (2.654-98.815)	0.003	1.323 (0.015-120.608)	0.903

AKI: acute kidney failure, CKD: chronic kidney disease, HR: hazard ratio, PHO: primary hyperoxaluria

## Discussion

This monocentric cohort study from a Tunisian nephrology department aimed to describe the prognostic features of adult HUL, to determine the incidence of progression of CKD and to identify factors influencing renal survival. We included 73 patients, of whom 60.3% (n=44) were males. The mean age at the onset of urolithiasis symptomatology was 19.15± 15.57 years. The causes were cystinuria, hereditary tubulopathies and primary hyperoxaluria (PH), in 31, 24 and 18 cases, respectively. The delay in the etiological diagnosis was over 5 years in 41.1% (n=30). After a follow-up of an average period of 16 years, the annual incidence of ESRD was 0.94% and the annual decline in glomerular filtration rate was 3.5 ml/min. The multivariate study retained only PH as an independent factor associated with the occurrence of ESRD, with an adjusted hazard ratio of 10.52, 95% CI: 1.664-66.507; p = 0.012. Primary hyperoxaluria had the highest annual incidence of ESRD during follow-up (3.42% vs. 0.46% for tubulopathies). The annual decline in glomerular filtration rate in PH was 5.73 ml/min, vs. 2.9 ml/min in cystinuria and 2.67 ml/min in tubulopathies. Our study is an extension of a previous descriptive study carried out in our department of nephrology [2]. Major methodological differences can be found between the two studies, this study being analytical and including more patients by extending the study period. The extended follow-up duration for a subset of our patients, particularly those referred from paediatric departments, enabled us to assess the long-term progression of HUL and to determine renal survival rates.

Most of these patients originated from our hospital's paediatric department and were previously documented in the study conducted by Boussetta *et al*. [4]. In this study, we also calculated the annual decline in eGFR, which highlights the rapid decline in renal function during these diseases, especially PH. In Tunisia, PH is frequent and responsible for 13% of CKD in children versus 0.3% in Europe and 0.7% in North America [5]. Patients with PH1 are more likely to have CKD at diagnosis and to develop CKD during follow-up. In our study, PH was an independent factor for progression to ESRD and it has been reported that PH1 has the worst renal prognosis with a renal survival of 27% at 30 years, compared with 92% and 95% for PH types 2 and 3, respectively [6]. Primary hyperoxaluria had the highest annual incidence of ESRD during follow-up (3.42%) and the fastest annual decline in GFR. Renal survival in PH was significantly lower than in other aetiologies. None of our cystinuria patients experienced ESRD. While renal failure has been observed in 17 to 28% of cystinuria patients, the progression to ESRD is infrequent, especially when patients are under careful monitoring [7]. The renal prognosis during HT depends on the aetiology. The prognosis is good during distal tubular acidosis and is affected in patients with familial hypomagnesemia with hypercalciuria and Dent's disease [8]. The strengths of our study were the relevance and originality of the topic, which focused on rare and often underrecognized causes of urinary stone disease and studies, and the extended follow-up duration for a subset of our patients enabled us to assess the long-term progression of HUL and to determine renal survival rates. However, our study also had limitations that should be considered: its monocentric and retrospective approach, resulting in some missing data and the genetic analysis, which focused only on AGXT gene mutations for PH1 and was performed only in 4 cases for HT. Moreover, there was a presence of selection bias. To elaborate, out of the total, 23 patients were directed from paediatric nephrology department for follow-up within an adult-care setting, while 9 patients had already existing chronic renal failure.

## Conclusion

Hereditary urolithiasis (HUL) in Tunisia is mainly due to cystinuria, hereditary tubulopathies, and primary hyperoxaluria (PH). A diagnostic delay exceeding five years was frequent, and PH was independently associated with an increased risk of progression to ESRD. The limited use of genetic testing, restricted to PH1 and a few cases of tubulopathies, underscores the urgent need to expand local genetic capabilities to enable earlier and more accurate diagnosis. Beyond etiological characterization, our findings highlight the importance of early metabolic evaluation and family screening, particularly in consanguineous populations, to improve renal outcomes. Strengthening multidisciplinary collaboration between nephrologists, urologists, geneticists, and pediatricians is essential to reduce diagnostic delays and slow progression to chronic kidney disease.

### What is known about this topic

- Hereditary urolithiasis (HUL) encompasses a group of rare genetic disorders causing recurrent kidney stones and progressive renal damage;
- Its true prevalence is underestimated due to underdiagnosis and limited access to genetic testing, particularly in low- and middle-income countries;
- Consanguinity significantly increases the burden of HUL, especially in North African populations; primary hyperoxaluria (PH) is one of the most severe forms, often leading to early-onset and recurrent stones and accelerated progression to chronic kidney disease when diagnosis and treatment are delayed.

### What this study adds

- This is the first long-term Tunisian cohort assessing renal survival in patients with hereditary urolithiasis over more than three decades;
- It provides real-world data on the incidence and predictors of end-stage renal disease (ESRD) in a setting where genetic testing remains limited;
- The study identifies primary hyperoxaluria (PH) as the major determinant of ESRD, emphasizing the crucial role of early etiological and genetic diagnosis in improving renal outcomes; by quantifying the rate of renal function decline across different HUL types, it highlights the urgent need to develop local genetic screening programs and to implement family-based prevention strategies in consanguineous populations.
